# Energy Transfer from Photosystem I to Thermally Reduced Graphene Oxide

**DOI:** 10.3390/ma11091567

**Published:** 2018-08-30

**Authors:** Karolina Sulowska, Kamil Wiwatowski, Piotr Szustakiewicz, Justyna Grzelak, Wiktor Lewandowski, Sebastian Mackowski

**Affiliations:** 1 Institute of Physics, Faculty of Physics, Astronomy, and Informatics, Nicolaus Copernicus University, Grudziadzka 5, 87-100 Torun, Poland; sulowska@fizyka.umk.pl (K.S.); kamilw@doktorant.umk.pl (K.W.); justynag@fizyka.umk.pl (J.G.); 2 Department of Chemistry, University of Warsaw, Pasteura 1, 02-093 Warsaw, Poland; p.szustakiewicz@student.uw.edu.pl (P.S.); wlewandowski@chem.uw.edu.pl (W.L.)

**Keywords:** energy transfer, reduced graphene oxide, protein, fluorescence microscopy

## Abstract

The energy transfer from photosynthetic complex photosystem I to thermally reduced graphene oxide was studied using fluorescence microscopy and spectroscopy, and compared against the structure in which monolayer epitaxial graphene was used as the energy acceptor. We find that the properties of reduced graphene oxide (rGO) as an energy acceptor is qualitatively similar to that of epitaxial graphene. Fluorescence quenching, which in addition to shortening of fluorescence decay, is a signature of energy transfer varies across rGO substrates and correlates with the transmission pattern. We conclude that the efficiency of the energy transfer depends on the number of rGO layers in the flakes and decreases with this number. Furthermore, careful analysis of fluorescence imaging data confirms that the energy transfer efficiency dependence on the excitation wavelength, also varies with the number of rGO flakes.

## 1. Introduction

Graphene, for over a decade, has cemented its place as one of the most studied materials [1]. Since 2004, when it was first obtained by mechanical exfoliation, its extraordinary properties have inspired many research groups to first of all probe and understand the genuine properties of this one-atom thin material [2,3]. On the other hand, graphene and its derivatives have quickly become components of hybrid systems, assembled in order to synergistically exploit their properties. These include exceptionally high electronic and thermal conductivity, mechanical strength, unusual electronic structure and optical transmittance, impermeability to gases, and many others [4], yielding applications in photovoltaics [5,6], optoelectronics [7,8], sensorics [9,10], etc. 

The key property of graphene, that impacts its electronic and optical character, is a zero bandgap energy structure accompanied with linear dispersion near the Brillouin zone corners. Full occupation of the valence band and an empty conduction band results in unique electronic absorption of graphene, which on the one hand is rather high (2.3% absorption of incident light [11]), and, in addition, shows no wavelength dependence within the visible spectral range. Consequently, from the point of view of hybrid assemblies where the energy transfer is exploited, graphene can be utilized as an energy acceptor, but due to the absence of any fluorescence emission, any impact of the energy transfer can only be evaluated and quantified using the properties of energy donors [12]. Among many examples, energy transfer to graphene has been observed for a variety of materials, including organic molecules [13,14], semiconductor nanocrystals [15,16], and photosynthetic complexes [17,18,19]. 

In essentially all cases, the efficiency of the energy transfer to graphene was very high, typically in the order of 90% or more, as evidenced by strong quenching of fluorescence intensity accompanied with shortening of fluorescence decays. In the case of photosynthetic complexes, where pigments are embedded in a protein scaffold, these values were slightly lower [20]. In an experiment where single CdSe/ZnS nanocrystals were deposited on graphene with a varied number of layers it has been shown [15], based on fluorescence intensity estimation, that the efficiency of the energy transfer increases with the number of graphene layers. However, measurements involving time-resolved fluorescence studies have led to the opposite conclusion [21]. The combination of high electron concentration and excellent energy acceptor properties in graphene, yields the unique dependence of the energy transfer efficiency on the excitation energy [20], as demonstrated for photosynthetic complexes deposited on graphene. In particular, it has been shown that excitation at 405 nm leads to a much faster energy transfer compared to the excitation at 640 nm. 

One graphene derivative is graphene oxide [22], which can be obtained using chemical methods, frequently in solution. Graphene oxide (GO) is a fluorescent insulator, as opposed to graphene, which is a non-emitting conductor. GO is an ideal precursor for the synthesis of reduced GO (rGO), also called chemically derived graphene. The GO to rGO reduction process achieves electrical and thermal conductivity, while diminishing any fluorescence. In contrast to graphene, rGO can be dispersed in water, and the preparation of rGO flakes in a solution makes it feasible to attach various functional groups on their surface [23,24].

While similarities between graphene and rGO are reflected in their role as energy acceptors, in contrast to graphene, rGO is mostly used as a fluorescence quencher in solutions, instead of in layer-by-layer geometries. It has also been demonstrated that incorporating rGO leads to fluorescence quenching of polymers [22,25], quantum dots [26,27], dye-labeled aptamers [28], and photosynthetic complexes [29]. 

In this work, we study the energy transfer in hybrid nanostructures composed of photosystem I deposited on both epitaxial graphene and thermally reduced GO. For both substrates, we observe strong quenching of fluorescence accompanied with shortening of fluorescence decay times. Both these observations confirm the emergence of the energy transfer from photosystem I to the respective graphene substrate. By correlating fluorescence intensity with the decay time, we show that the efficiency of the energy transfer from photosystem I to rGO depends, in a qualitatively similar way as for graphene, on the number of rGO layers. In addition, comprehensive fluorescence microscopy imaging with varied excitation wavelengths indicates that the efficiency of the energy transfer from photosystem I to rGO depends on not only the excitation wavelength, but also on the number of rGO layers. 

## 2. Materials and Methods

We used photosystem I (PSI) isolated from algae *Cyanidioschyzon merolae* as the energy donor. Isolation was carried out as described previously [30]. The initial concentration of chlorophylls in PSI was 1.2 mg/mL, by denaturizing the protein in methanol and measuring the absorption spectrum of chlorophylls. Substrates containing monolayer graphene were purchased from ACS Materials (Pasadena, CA, USA). They were prepared on 22 × 22 mm glass coverslips by transferring chemical vapor deposition—grown monolayer graphene. The quality of the graphene substrate was checked with Raman spectroscopy (Figure S1). 

The reduced graphene oxide (rGO) was synthesized using a modified Hummers method [31]. Briefly, 1 g of powdered graphite (20 µm mesh size, Sigma Aldrich, (Saint Louis, MO, USA) was dispersed in 85:15 sulfuric/phosphoric acid mixture. Then, the reaction vessel was placed in an ice bath and 6 g of potassium permanganate was added in portions under vigorous stirring. Fifteen minutes after the addition of the last portion of KMnO_4_ the reaction mixture was brought to 50 °C. To support the exfoliation process, 2 h later the reaction mixture was placed for 2 h in an ultrasonic bath and then left at 50 °C under mild stirring overnight (12 h). Then, the mixture was poured into a glass beaker with ice cubes and hydrogen peroxide was slowly added to reduce the remaining KMnO_4_ (until the solution turned light yellow). The crude product was collected by centrifugation (10 min, 5000 rpm) and washed in consecutive dispersion/centrifugation cycles with concentrated sodium bicarbonate solution and copious amounts of water. The process was repeated until the supernatant pH was neutral. Finally, the obtained graphene oxide was re-dispersed in water to 1 mg/mL concentration. Successful exfoliation of graphene oxide sheets was confirmed using scanning electron microscope (SEM) imaging of a drop-casted material (Figure S2); this measurement also suggests that GO flakes of different thickness were obtained. Quality of the materials (uniformity of size and exfoliation) was further confirmed by observation of the liquid crystalline properties of the graphene oxide aqueous dispersion (Figure S3) [32].

To prepare the samples of rGO, the prepared aqueous dispersions of GO were spin-coated onto glass coverslips (30 s, 2000 rpm) and dried with nitrogen. The material was thermally reduced by keeping the substrates at 250 °C for 2 h under argon atmosphere [33]. During heating, the thin films changed color from light brown to black indicating the successful production of rGO [34], as shown in Figure S4, where coverslips with GO and rGO materials are displayed.

SEM imaging was performed using a LEO 435VP (Zeiss, Oberkochen, Germany) scanning electron microscope. The confirmation of liquid crystalline character of graphene oxide aqueous dispersion has been achieved by placing the sample between crossed polarizers and shaking the material to induce water flow.

The absorption spectra of the PSI solution and rGO sample were measured using a Carry 50 UV-Vis spectrophotometer (Varian, Palo Alto, CA, USA). The excitation and emission spectra of the PSI solution (diluted by a factor of 100 from the stock solution) were acquired with FluoroLog 3 spectrofluorometer (HORIBA Jobin Yvon Inc., Edison, NJ, USA) with a Xenon lamp and thermoelectrically cooled photomultiplier tube used for excitation and detection, respectively. The spectral resolution for both excitation and detection pathways was 0.5 nm. 

Three samples were fabricated: A layer of PSI-containing PVA polymer spin-coated on the rGO substrate, a layer of PSI-containing PVA polymer spin-coated on the graphene substrate, and a reference sample with PSI-containing PVA polymer spin-coated on a glass substrate. The PSI solution was diluted by a factor of 1000 from the stock solution. Such a low concentration of the protein combined with PVA polymer (0.05%) results in very thin layers on both the glass coverslips and the graphene and rGO substrates (less than 50 nm), which are essential for observing the effects associated with the energy transfer from PSI. 

The optical properties of the hybrid nanostructures were studied by means of fluorescence microscopy and spectroscopy using wide-field microscopy and confocal microscopy. The main experiment was carried out using a home-built fluorescence confocal microscope with the capabilities of spectrally- and time-resolved fluorescence detection. The sample was placed on a piezoelectric translation stage, the movement of which was correlated with a readout from a SPCM-16 detector (Perkin Elmer, Waltham, MA, USA). The excitation light of 485 nm (repetition rate of 20 MHz, average power of 12 µW) was used to excite the fluorescence of PSI. The laser beam was focused by a 60× oil-immersion objective (Nikon, Minato, Tokyo, Japan) with a numerical aperture of 1.49. The same objective was used for collecting fluorescence from the sample. In order to spectrally select PSI emissions, we used a longpass filter (HQ 665LP, Chroma, Bellows Falls, VT, USA) and a bandpass filter (HQ 675/20, Chroma, Bellows Falls, VT, USA). Fluorescence spectra were detected using a combination of Andor iDus DV 420A-BV CCD detector (Oxford Instruments Group, Belfast, Northern Ireland, UK) coupled with an Amici prism. Time-resolved measurements were performed by a time-correlated single photon counting technique using an SPC-150 module (Becker & Hickl, Nunsdorfer Ring, Berlin, Germany) with a fast avalanche photodiode (idQuantique id100-20, SK Telecom, Carouge, Genève, Switzerland) as a detector.

A Nikon Eclipse Ti-U microscope (Minato, Tokyo, Japan) equipped with an Andor iXon Du-888 EMCCD detector (Oxford Instruments Group, Belfast, Northern Ireland, UK) was used for wide-field fluorescence imaging. Three excitation wavelengths were used to illuminate the samples (405 nm, 480 nm, and 535 nm) with the same power of 100 µW, and the region of illumination was about 90 × 90 microns. An oil-immersion microscope objective with a magnification of 100× (Plan Apo, Nikon, Minato, Tokyo, Japan) and a numerical aperture of 1.4 was used. The fluorescence signal was extracted using a combination of a dichroic mirror T650lxpr (Chroma, Bellows Falls, VT, USA), longpass filter FELH650 (Thorlabs, Newton, NJ, USA), and a bandpass filter FB670/10 (Thorlabs, Newton, NJ, USA). The electron multiplying gain and acquisition time were 700 and 1 seconds, respectively. 

## 3. Results

**Figure 1:**
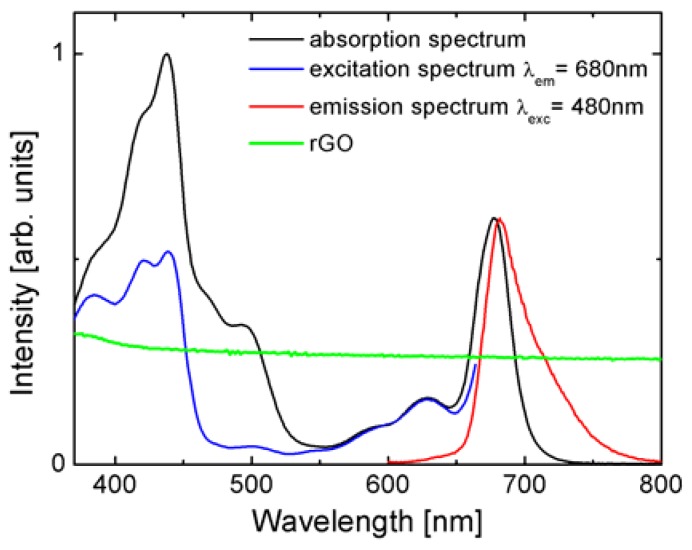
Absorption (black line), fluorescence (red), and fluorescence excitation (blue line) spectra of photosystem I (PSI) complexes in a buffer solution. Green line corresponds to the absorption of the reduced graphene oxide (rGO) layer.

Before studying the energy transfer in hybrid nanostructures composed of Photosystem I deposited on both epitaxial graphene and thermally reduced GO, we first determined the optical properties of the constituents. In Figure 1 we compare the optical properties of PSI complexes in the solution with the absorption of rGO layer/thin film. The absorption of PSI (black line in Figure 1) spans over the whole visible range with the bands corresponding to different pigments forming the PSI structure [30]. The Soret band of Chl a shows strong absorption bands in the UV and blue regions. Another band is placed in the red and near the IR range and is associated with the Qy band of Chl a. The presence of carotenoids is evidenced by the weaker absorption centered around 500 nm. The emission band of PSI appears at 678 nm (red line in Figure 1) and originates from the recombination of the first excited state of Chl a. Furthermore, we observe a second, less-intense band from red Chls. The fluorescence excitation spectrum (blue line in Figure 1) follows the absorption spectrum only for wavelengths longer than 550 nm, i.e., in the chlorophyll region. On the other hand, for shorter wavelengths the similarity is much less evident, although some of the bands can be distinguished. The differences between absorption and fluorescence excitation are common for photosynthetic complexes, as not all of the pigments comprising such a complex participate in the energy transfer directly to the chlorophylls that feature fluorescence emission. Importantly, for the experiments described in this work, the emission of PSI can be excited with wavelengths from 405 nm to 535 nm. The absorption of the rGO layer shown in green is uniform over the whole visible range, which is analogous to the behavior found for graphene. 

The absorption of rGO was measured on a clean glass substrate (green line in Figure 1). The uniform, non-zero absorption throughout the visible confirms successful reduction of graphene oxide [35]. In additionS, it shows that the optical properties of rGO are similar to graphene [11], with the only difference being that within our sample we probe spots of different thickness (number of layers) of reduced graphene oxide.

The emergence of the energy transfer from a donor, in our case the PSI complex, to an acceptor, in our case graphene or rGO, can be observed as a decrease of the fluorescence emission of the PSI complexes associated with the shortening of fluorescence decay thereof [12,15,19]. In order to study these effects, confocal fluorescence microscopy was applied. 

**Figure 2:**
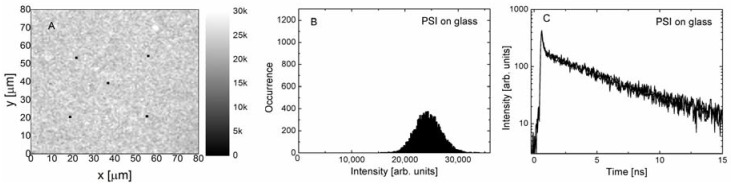
(**A**) Fluorescence intensity map measured for photosystem I (PSI) complexes on a glass substrate using the excitation wavelength of 485 nm. (**B**) Histogram of fluorescence intensity extracted from the map. (**C**) Fluorescence intensity decays measured for the locations marked by spots in (A).

In Figure 2 we collect the results obtained for PSI complexes embedded in PVA deposited on a glass coverslip. This sample is our reference. The experiment was carried out using the excitation wavelength of 485 nm. The fluorescence emission map of PSI shown in Figure 2A is very homogeneous, and as indicated with the histogram of emission intensities (Figure 2B), the average value is about 25 kpcs, and the distribution is of Gaussian shape. Accordingly, fluorescence decay curves measured for the five spots across the map, as marked in Figure 2A, are essentially identical, and similar to those measured previously for this photosynthetic complex [36]. The decays are bi-exponential, with the average decay time of PSI-LHCI equal to 1.65 ns (Figure 2C). The results of fluorescence imaging and spectroscopy confirm that PSI complexes embedded in the PVA layer form uniform, high-quality layers on a glass substrate. In addition, the optical properties of the PSI complexes in a layer are similar to those in the buffer solution.

**Figure 3:**
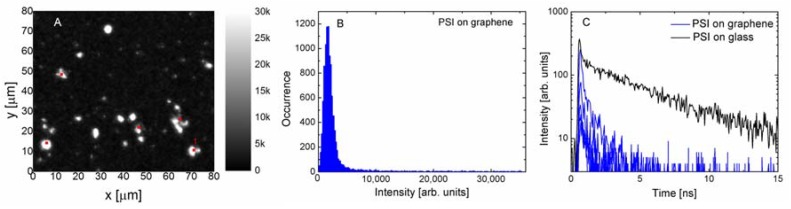
(**A**) Fluorescence intensity map measured for PSI complexes deposited on a monolayer graphene using the excitation wavelength of 485 nm. (**B**) Histogram of fluorescence intensity extracted from the map. (**C**) Fluorescence intensity decays measured for PSI complexes on monolayer graphene (blue lines) for the spots indicated in (A) compared with the reference transient (black line).

Depositing emitters on graphene often results in strong energy transfer [19], leading to fluorescence quenching of emission. In Figure 3 we show the results obtained for PSI complexes deposited on a monolayer graphene. Although the excitation and detection conditions were identical in the case of the structure where PSI complexes were deposited on a glass substrate, the fluorescence image (Figure 3A) is radically different. Most of the measured emissions have very low intensities and only a few bright spots can be seen. This result is qualitatively similar to previous data obtained for simpler photosynthetic complexes [20,21], where strong fluorescence quenching of emission was also observed. The bright spots were attributed to cracks in the graphene layer, where an accumulation of protein may take place. The histogram displayed in Figure 3B confirms the qualitative description of the data, the intensities are approximately 10 times less than in the case of reference. In fact, it should be noted that the intensity is somewhat higher, as it is required to account for the absorption of the graphene layer itself, which amounts to 2.3%. It affects both the excitation laser and the fluorescence emission, but even including this effect would not account for the differences in emission intensity. The unambiguous evidence of emerging energy transfer from PSI to graphene comes from the results shown in Figure 3C, where the fluorescence decay curves are compared against the reference. We find that the decays are substantially shorter upon depositing the PSI complexes on graphene. Moreover, the efficiency of the energy transfer seems to be rather uniform, which can be inferred from both the narrow width of the intensity distribution, as well as from the similarity between the decay curves measured for various locations across the sample. The average fluorescence lifetime of PSI complexes on monolayer graphene is 0.93 ns, which proves efficient energy transfer from the PSI to graphene. 

The sample containing thermally reduced graphene oxide is formed by randomly located rGO flakes with different thicknesses. It thus allows probing of the effect of the number of rGO layers on the efficiency of the energy transfer. Previously it has been shown that depending on the actual experimental configuration the energy transfer efficiency can either increase or decrease with the number of graphene layers [15,21]. 

**Figure 4:**
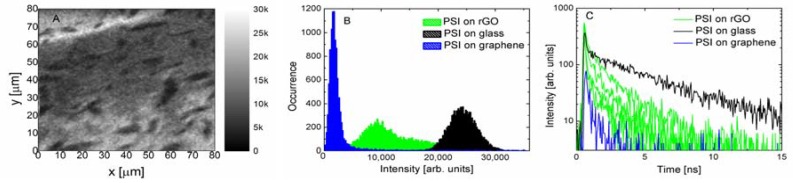
(**A**) Fluorescence intensity map measured for the PSI complexes deposited on thermally reduced graphene using the excitation wavelength of 485 nm. (**B**) Histogram of fluorescence intensity extracted from the map (green) compared to the data obtained for PSI complexes deposited on a glass substrate (black) and a monolayer graphene (blue). (**C**) Fluorescence intensity decays measured for PSI complexes on thermally reduced graphene oxide (green lines) for several spots on the map shown in (**A**) compared both with the reference (black line) and the decay measured for PSI on monolayer graphene (blue line).

In Figure 4 the results of the fluorescence obtained for the PSI complexes on the rGO substrate are displayed. The fluorescence image is rather inhomogeneous, featuring similar percentages of bright and dark areas, which are visualized with a histogram in Figure 4B. In fact, the intensities measured for the PSI complexes on rGO fit between the values measured for PSI on the glass substrate and PSI on monolayer graphene. Again, similar to the results measured for monolayer graphene, one has to keep in mind that the actual intensities are slightly different, being modified by the absorption of the laser light and the fluorescence by the rGO layers. However, for the rGO substrate this is a rather complex issue. Importantly, in the case of this structure, measuring fluorescence dynamics provides evidence of the energy transfer. The decays measured for several locations across the sample containing the PSI complexes on the rGO substrate (green lines) are compared with the data acquired for the PSI on graphene (blue line) and on the glass substrate (black line) (Figure 4C). Analogously, as in the case of fluorescence intensities, the fluorescence dynamics of the PSI complexes on rGO fits between the behavior observed for the two boundary cases. As it is natural to expect that decay curves were collected for regions with a different number of rGO layers in a flake, the spread of decay times suggests that, similar to the multilayer graphene substrates, the efficiency of the energy transfer also depends on the number of rGO flakes. 

**Figure 5:**
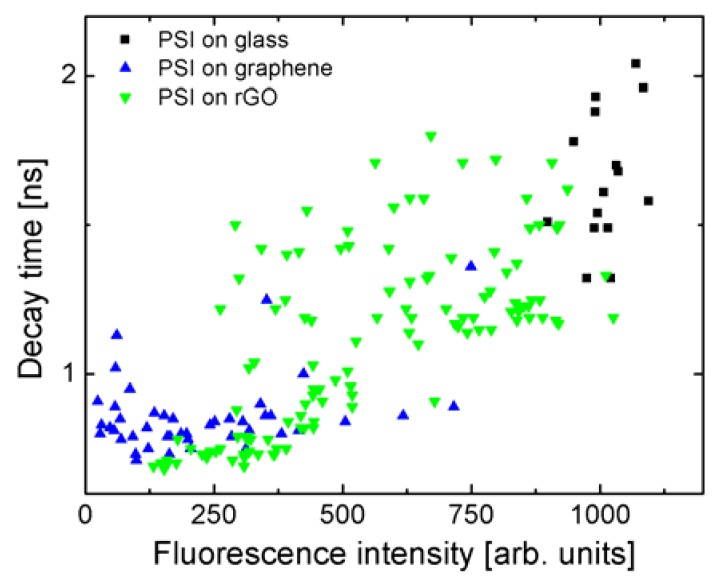
Correlation plot displaying the relation between integrated fluorescence intensity and the decay time, for PSI complexes deposited on glass (black squares), monolayer graphene (blue triangles), and rGO (green triangles) substrates.

The correlation between the fluorescence intensity and the decay times is shown in Figure 5 for each sample—experimental points extracted for many maps for each of the substrates are shown. Fluorescence intensities were extracted by integrating decays curves. The values measured for the reference, where PSI complexes are deposited on a glass substrate (black squares) are merged in well-defined areas, with fluorescence intensities around 1000 units and decay times around 1.75 ns. Similarly, for PSI on monolayer graphene (blue triangles), the values of decay times and fluorescence intensities are grouped in a rather small region, with decay times around 0.9 ns, and intensities around 200 units. The relation between fluorescence intensities extracted from the fluorescence decay curves is identical to the one measured using fluorescence imaging. Importantly, the data points obtained for PSI on rGO are distributed between the two other samples (green triangles). Furthermore, we can see a correlation between both values, measured independently in a single experiment. Namely, the shorter the decay time, the lower the fluorescence intensity of the PSI emission. This result shows that the efficiency of the energy transfer, measured by the decay time, depends on the number of rGO flakes. From the comparison between all three studied structures, we can conclude that an increase in the number of rGO layers results in a reduction of the energy transfer efficiency, as shown previously for multilayer CVD graphene [21].

Another aspect of the energy transfer in graphene-based hybrid nanostructures, concerns the dependence of the energy transfer efficiency on the excitation wavelength [20]. It has been shown that excitation with blue light results in a much faster energy transfer rates compared to excitation with red light. The structure, where the PSI complexes are deposited on the rGO substrate, where the number of rGO layers varies across the sample, offers a unique possibility to explore this effect in a greater detail. 

**Figure 6:**
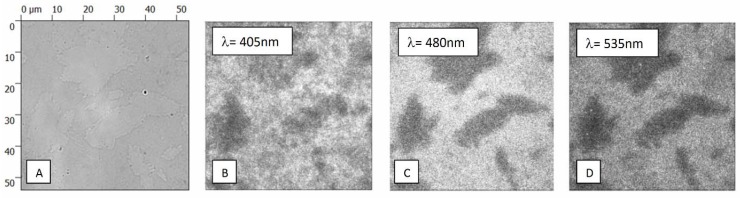
(**A**) Transmission microscopic image of the sample with PSI complexes deposited on rGO. (**B**–**D**) Fluorescence maps acquired for the same sample area using three different excitation wavelengths of 405 nm, 480 nm, and 535 nm.

In Figure 6 we present a series of fluorescence images obtained for the PSI complexes deposited on rGO excited with wavelengths of 405 nm, 480 nm, and 535 nm. The studied region of the sample is the same for all of the excitation wavelengths. In addition, we also show a transmission image, where islands of various sizes can be seen. We attribute these to rGO flakes with different thicknesses. Importantly, fluorescence images reveal a similar pattern, with bright and dark regions, the shapes of which are identical with the contrasts seen in the transmission image. In particular, for the excitation wavelength of 405 nm the fluorescence intensity map is the most intense, although the islands are the least visible, the contrast is weak. This observation suggests that the dependence of the excitation energy on the efficiency of the energy transfer to rGO depends on the number of rGO layers.

**Figure 7:**
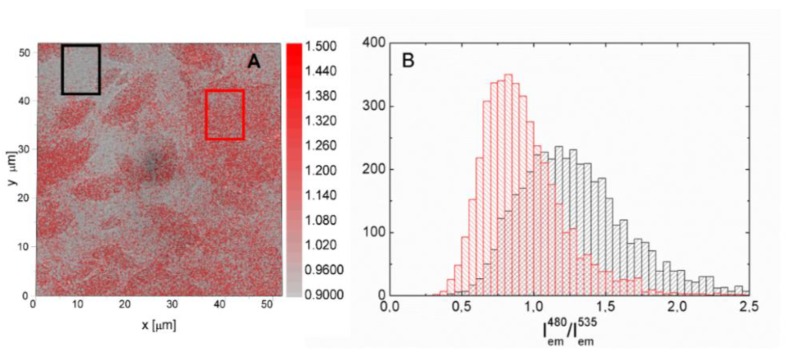
(**A**) Map obtained by dividing fluorescence intensity maps of PSI on rGO measured for the excitations of 480 nm and 535 nm (I_em_^480^)/(I_em_^535^) superimposed over the transmission image. (**B**) Histograms of (I_em_^480^)/(I_em_^535^) ratio obtained for two selected but homogenous regions of the map shown in (**A**).

In order to prove this dependence, we divided fluorescence intensity maps measured for two excitation wavelengths of 480 nm and 535 nm. The result of this procedure is shown in Figure 7A, where the map of intensity ratio is superimposed on the transmission image. It can be seen that the values of the intensity ratio correlate with the grey scale map of transmission. If the excitation wavelength dependence of the efficiency of the energy transfer is independent of the number of rGO layers, the map obtained by dividing the fluorescence map should be uniform. This is clearly not the case for the result displayed in Figure 7 and in Figure S5. Confirmation comes from the comparison shown in Figure 7B, where histograms extracted from two areas with pretty uniform values of (I_em_^480^)/(I_em_^535^) ratio are compared. Both the maximum values of the intensity ratio as well as the broadening of the distribution is different between these two areas and this result bears a general character. Consequently, the combination of fluorescence imaging with the ability to fabricate thermally reduced graphene oxide with flake sizes in the range of tens of microns, allows us to conclude that the dependence of the efficiency of the energy transfer to rGO on the excitation energy is in addition dependent on the number of rGO layers. 

## 4. Conclusions

The results of the fluorescence imaging and spectroscopy obtained for a hybrid nanostructure composed of Photosystem I and thermally reduced GO indicate that the properties of rGO as an energy acceptor are qualitatively similar to those of epitaxial graphene. In particular, strong fluorescence quenching is observed, with the efficiency of this quenching being dependent on the number of rGO layers in the flakes. Careful analysis of fluorescence imaging data confirms not only that the efficiency of the energy transfer to rGO depends on the excitation wavelength, but also that this dependence changes with the number of rGO flakes.

## Supplementary Materials

The following are available online at http://www.mdpi.com/1996-1944/11/9/1567/s1, Figure S1: Raman spectrum measured for the graphene substrate used in the experiments, Figure S2: SEM image of a dropcasted graphene oxide dispersion, Figure S3: Photography of a GO aqueous dispersion. The sample was illuminated from the back with a polarized light source (direction of the polarization is indicated by the arrow on the right). Half of the sample was covered with an additional polarizer as indicated on the left. Bright areas in the two-crossed polarizers area evidence liquid crystalline character of the sample, Figure S4: Glass coverslips with spin coated graphene oxide before (one the left) and after (on the right) thermal reduction, Figure S5: Transmission map compared with the map of fluorescence intensity ratio measured for PSI complexes on rGO excited with 405 nm and 535 nm excitation wavelengths.
