# Supplementary material for: Energy Transfer from Photosystem I to Thermally Reduced Graphene Oxide
Source: Materials (Basel). 2018 Aug 30;11(9):1567. doi: 10.3390/ma11091567 (PMC6164758; doi:10.3390/ma11091567)
Supplement: Supplementary file 1 [file materials-11-01567-s001.pdf]

Supplementary Information: Energy transfer from Photosystem I to thermally reduced graphene oxide

Karolina Sulowska[1], Kamil Wiwatowski[1], Piotr Szustakiewicz[2], Justyna Grzelak[1], Wiktor Lewandowski[2], Sebastian Mackowski[1]

[1]Institute of Physics, Faculty of Physics Astronomy and Informatics, Nicolaus Copernicus University, Grudziadzka 5, 87-100 Torun, Poland
[2]Department of Chemistry, University of Warsaw, Pasteura 1, 02-093 Warsaw, Poland

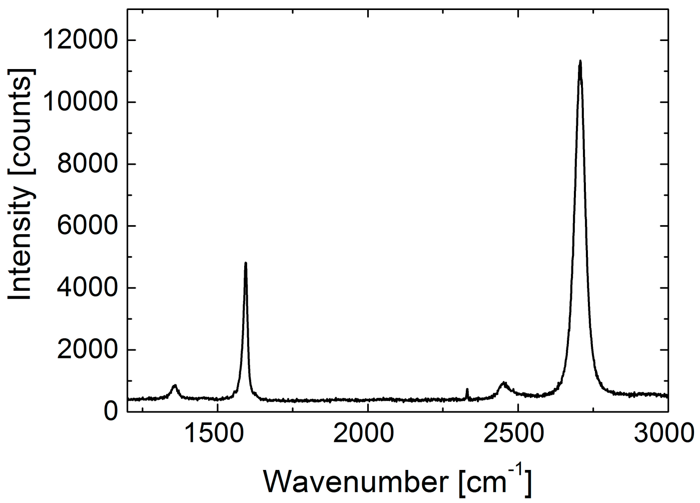

Figure S1. Raman spectrum measured for the graphene substrate used in the experiments.

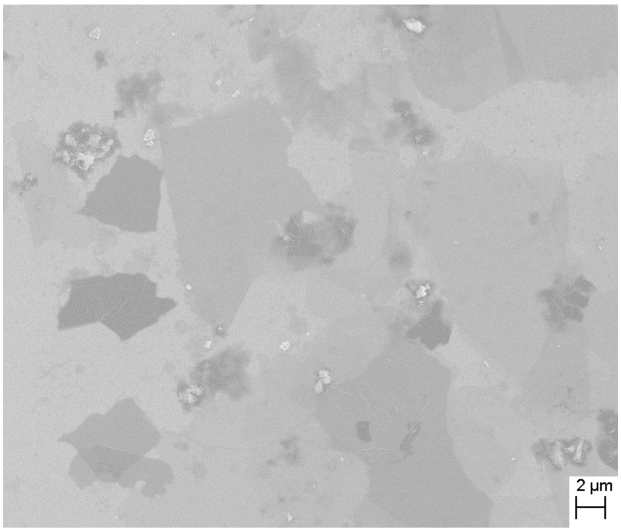

Figure S2. SEM image of a dropcasted graphene oxide dispersion.

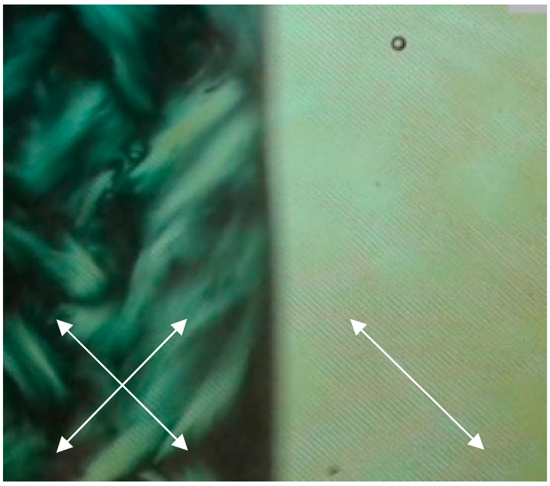

Figure S3. Photography of a GO aqueous dispersion. The sample was illuminated from the back with a polarized light source (direction of the polarization is indicated by the arrow on the right). Half of the sample was covered with an additional polarizer as indicated on the left. Bright areas in the two-crossed polarizers area evidence liquid crystalline character of the sample.

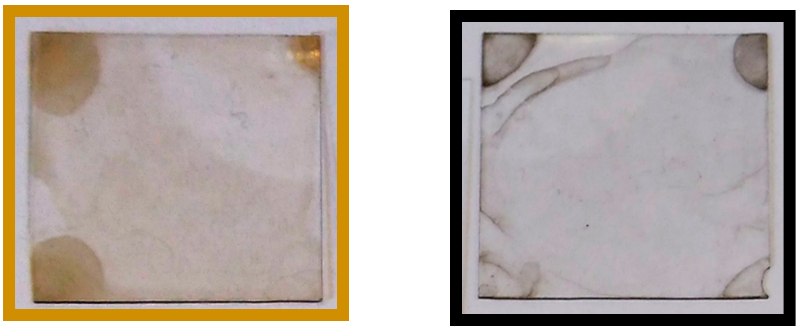

Figure S4. Glass coverslips with spin coated graphene oxide before (one the left) and after (on the right) thermal reduction.

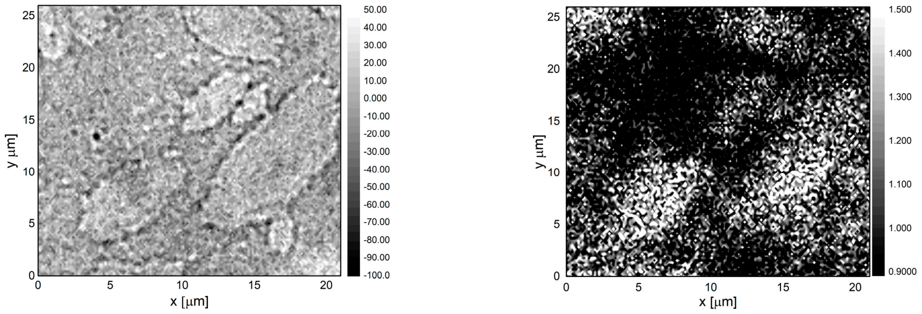

Figure S5. Transmission map compared with the map of fluorescence intensity ratio measured for PSI complexes on rGO excited with 405 nm and 535 nm excitation wavelengths.
